# Genome-wide identification of the expansin gene family in netted melon and their transcriptional responses to fruit peel cracking

**DOI:** 10.3389/fpls.2024.1332240

**Published:** 2024-01-23

**Authors:** Yanping Hu, Yuxin Li, Baibi Zhu, Wenfeng Huang, Jianjun Chen, Feng Wang, Yisong Chen, Min Wang, Hanggui Lai, Yang Zhou

**Affiliations:** ^1^ School of Tropical Agriculture and Forestry (School of Agricultural and Rural Affairs, School of Rural Revitalization), Key Laboratory for Quality Regulation of Tropical Horticultural Crops of Hainan Province, Haikou, China; ^2^ Sanya Institute, Hainan Academy of Agricultural Sciences, Sanya, China; ^3^ The Institute of Vegetables, Hainan Academy of Agricultural Sciences, Key Laboratory of Vegetable Biology of Hainan Province, Hainan Vegetable Breeding Engineering Technology Research Center, Haikou, China

**Keywords:** expansins, fruit cracking, gene expression, co-expression network, netted melon

## Abstract

**Introduction:**

Fruit cracking not only affects the appearance of netted melons (*Cucumis melo* L. var. reticulatus Naud.) but also decreases their marketability.

**Methods:**

Herein, to comprehensively understand the role of expansin (EXP) proteins in netted melon, bioinformatics methods were employed to discover the *EXP* gene family in the melon genome and analyze its characteristic features. Furthermore, transcriptomics analysis was performed to determine the expression patterns of melon *EXP* (*CmEXP*) genes in crack-tolerant and crack-susceptible netted melon varieties.

**Discussion:**

Thirty-three *CmEXP* genes were identified. Chromosomal location analysis revealed that *CmEXP* gene distribution was uneven on 12 chromosomes. In addition, phylogenetic tree analysis revealed that *CmEXP* genes could be categorized into four subgroups, among which the EXPA subgroup had the most members. The same subgroup members shared similar protein motifs and gene structures. Thirteen duplicate events were identified in the 33 *CmEXP* genes. Collinearity analysis revealed that the *CmEXP* genes had 50, 50, and 44 orthologous genes with *EXP* genes in cucumber, watermelon, and *Arabidopsis*, respectively. However, only nine orthologous *EXP* genes were observed in rice. Promoter *cis*-acting element analysis demonstrated that numerous *cis*-acting elements in the upstream promoter region of *CmEXP* genes participate in plant growth, development, and environmental stress responses. Transcriptomics analysis revealed 14 differentially expressed genes (DEGs) in the non-cracked fruit peels between the crack-tolerant variety ‘Xizhoumi 17’ (N17) and the crack-susceptible variety ‘Xizhoumi 25’ (N25). Among the 14 genes, 11 were upregulated, whereas the remaining three were downregulated in N17. In the non-cracked (N25) and cracked (C25) fruit peels of ‘Xizhoumi 25’, 24 DEGs were identified, and 4 of them were upregulated, whereas the remaining 20 were downregulated in N25. In the two datasets, only *CmEXPB1* exhibited consistently upregulated expression, indicating its importance in the fruit peel crack resistance of netted melon. Transcription factor prediction revealed 56 potential transcription factors that regulate *CmEXPB1* expression.

**Results:**

Our study findings enrich the understanding of the *CmEXP* gene family and present candidate genes for the molecular breeding of fruit peel crack resistance of netted melon.

## Introduction

Fruit peel directly affects the interaction between plants and the external environment and determines the fruit harvesting period and their ability to resist biotic or abiotic stress (Riglet et al., 2021). However, fruit cracking alters the mechanical properties and integrity of the peel, resulting in visual damage, decreased quality, significantly increased risk of pathogen and parasite infestation, shortened shelf life, and increased susceptibility to decay, thereby severely affecting the commercial value of fruits (Knoche and Lang, 2017; Jiang et al., 2019). As a common disorder, fruit cracking occurs in various horticultural crops, including apple (Opara, 1996), tomato (Emmons and Scott, 1997), pomegranate (Davarpanah et al., 2016), litchi (Wang et al., 2019), sweet cherry (Schumann and Knoche, 2020), and jujube (Liu et al., 2023). Both internal and external factors are involved in fruit cracking. Internal factors primarily include genetics and the intrinsic characteristics of the fruit, such as fruit size, shape, growth rate, moisture content, fruit peel properties, and fruit cracking-related gene expression (Khadivi-Khub, 2015; Yang et al., 2016). On the other hand, external factors primarily include growth environmental conditions, including temperature, light, and precipitation, and cultivation management practices, including irrigation, shading, and application of minerals and growth regulators (Dominguez et al., 2012). Moreover, cracking is associated with the loss of fruit hardness and cell wall integrity (Moctezuma et al., 2003; Cao et al., 2012).

Expansins (EXP) and Xyloglucan endotransglycosylase (XET) involved in cell wall relaxation are closely related to fruit softening and fruit cracking (Hayama et al., 2006; Asha et al., 2007). EXP, a nonenzymatic protein found in the cell wall of plants, plays a vital role in cell expansion. It breaks the hydrogen bonds between cellulose microfibrils and hemicelluloses, consequently regulating the relaxation degree between cell wall components and increasing cell wall flexibility (Feng et al., 2019). EXP was first identified in experiments on the “acid growth” of the cell wall (McQueen-Mason et al., 1992; Li et al., 1993). EXP is a large conserved gene family, with a relative molecular weight (MW) of approximately 26 kDa. It is divided into four subfamilies according to differences in gene sequences and structural composition: α-EXP (EXPA), β-EXP (EXPB), EXP-like A (EXLA), and EXP-like B (EXLB) (Kende et al., 2004). Advances in genome sequencing have resulted in the identification of *EXP* genes in numerous species, including *Arabidopsis thaliana* (Sampedro et al., 2006), rice (Sampedro et al., 2006), grape (Dal Santo et al., 2013), apple (Zhang et al., 2014a), corn (Zhang et al., 2014b), soybean (Zhu et al., 2014), tobacco (Ding et al., 2016), tomato (Lu et al., 2016), and cucumber (Liu et al., 2022a).

EXP plays an essential role in the development and growth of plants, including seed germination (Yan et al., 2014), root elongation (Noh et al., 2013), fruit ripening (Jiang et al., 2019), salt tolerance (Lv et al., 2013), and drought resistance (Chen et al., 2019). During seed softening, *LeEXP4* expression increases in tomato, and this gene may participate in seed coat softening and cell wall relaxation, thereby promoting seed germination (Chen et al., 2001). After silencing *LeEXP1*, the hardness of the tomato decreased, with the promotion of hemicellulose decomposition (Brummell et al., 1999). In pear fruits, the ethylene inhibitor 1-methylcyclopropene inhibited fruit softening and suppressed *PcEXP2*, *LeEXP3*, and *LeEXP5* expression, whereas an ethylene promoter induced their expression, indicating that these EXP proteins respond to ethylene induction and participate in fruit softening (Hiwasa et al., 2003). The analysis of the relationship between *MdEXPA3* expression in the pericarp and mesocarp of ‘Fuji’ apple and fruit cracking during fruit growth revealed that inducing *MdEXPA3* accumulation in the outer pericarp decreases the fruit’s sensitivity to cracking (Kasai et al., 2008). Xue et al. (2020) have reported that EXP proteins regulate water-induced tomato fruit cracking. The expression of *EXPA-like* genes is significantly higher in the fruit peels of the crack-resistant jujube variety ‘Wanzao 3’ than in the crack-susceptible jujube variety ‘Lifu Gongzao’ (Xin et al., 2021). Furthermore, simultaneously inhibiting polygalacturonase (PG) and *EXP* gene expression in tomato can decrease PG and EXP protein and water-soluble pectin contents in the fruit peel and increase cell wall thickness and hardness, thereby decreasing fruit cracking rate (Jiang et al., 2019). Therefore, EXP may play a vital role in fruit cracking regulation.

Netted melon (*Cucumis melo* L. var. reticulatus Naud.) is a type of thick-peeled melon belonging to the family Cucurbitaceae. The term ‘netted melon’ is used owing to the presence of net-like structures on the surface of its mature fruit (Sun et al., 2013). The netting is uniform and aesthetically pleasing, with sweet and juicy flesh, high sugar content, and a rich aroma. Furthermore, netted melon is resistant to storage and transportation, making it popular among consumers (Shi et al., 2015). At present, the cultivation area of netted melon exceeds 460,900 hectares annually in China (Chang et al., 2019). Netting degree is an important quality trait of netted melon. However, during netted melon production, adverse issues such as the uneven size of the fruit surface netting and uneven netting formation may occur. Under unfavorable environmental conditions, fruit cracking can occur at the site of netting formation, significantly affecting its commercial appeal. Many studies have reported that netting formation in netted melons is closely associated with environmental factors, including plant hormones, water, temperature, and light (Chen, 2010; Wang et al., 2011; Li et al., 2020). However, studies on the molecular mechanisms underlying the cracking resistance of netted melon are scarce. Herein, based on the genome data of melons, *EXP* genes associated with crack resistance were identified, and their features were characterized. Furthermore, using the transcriptome data of our laboratory, the *EXP* gene expression patterns in the mature fruit peels of crack-resistant and crack-susceptible varieties were elucidated. Our study findings provide important implications for exploring crack resistance-related genes in the fruit peels of netted melon and deciphering their molecular mechanisms.

## Materials and methods

### Genome-wide *EXP* gene identification in netted melon

The reference genome of melon was downloaded from GuGenDBv2 (http://cucurbitgenomics.org/v2/ftp/genome/melon/DHL92/v4.0/). DPBB_1 (PF03330) and Pollen_allerg_1 (PF01357), the Hidden Markov Model (HMM) profiles of two classical domains, were utilized to identify melon *EXP* genes by searching the putative *EXP* genes in the melon protein dataset using HMMsearch, and the E-value threshold was <10^−5^ (Jin et al., 2020). Then, the conserved domains were confirmed by submitting the obtained putative EXP protein sequences to Pfam (http://pfam.xfam.org/), Simple Modular Architecture Research Tool (http://smart.embl.de/smart/batch.pl), and Conserved Domain Database (https://www.ncbi.nlm.nih.gov/Structure/bwrpsb/bwrpsb.cgi). Thereafter, superfluous sequences and the predicted protein sequences without common domains were removed, and the candidate genes were ascribed as *Cucumis melo EXP*s (*CmEXP*s) and named according to their chromosomal positions. ExPASy (Expasy 3.0; http://web.expasy.org/protparam/) was used to elucidate the physical and chemical characteristics, including gene locations, MW, and theoretical isoelectric point (pI) of CmEXPs.

### Phylogenetic tree construction of *CmEXP* genes

EXPANSIN CENTRAL (http://www.personal.psu.edu/fsl/ExpCentral/, accessed on 10 July 2020) was utilized to obtain the *EXP* sequences from *Arabidopsis thaliana* and rice (*Oryza sativa*). Previous studies have identified the *EXP* genes from cucumber (*Cucumis sativus*) and watermelon (*Citrullus lanatus*) (Gao et al., 2020; Liu et al., 2022a). ClustalW was employed to align the EXP protein sequences of these five species. Then, the maximum likelihood method in MEGA X was employed to construct an unrooted phylogenetic tree, with pairwise deletion and 1000 bootstrap replicates. EvolView (https://evolgenius.info/evolview-v2) was utilized to process the resulting phylogenetic tree.

### Gene structure, conserved motif, and *cis*-element analyses of *CmEXP*s

The conserved motifs of CmEXP proteins were elucidated using the Multiple Em for Motif Elicitation (MEME) program (MEME Suite 5.3.3; http://meme-suite.org/tools/meme, accessed on 9 September 2022). The following parameters were used: maximum number of motifs of 10 and motif width of 6–100 amino acid residues. TBtools software was employed to extract the exon–intron data for *CmEXP*s from the coding and genome sequences of the related genes (Chen et al., 2020). The same software was utilized to envision the conserved motifs and gene structures.

TBtools was used to extract the promoter region, a 2.0 kb upstream sequence of the start codon, of each *CmEXP* gene, and to elucidate the *cis*-regulatory elements (CREs). The extracted sequences were then submitted to PlantCARE (http://bioinformatics.psb.ugent.be/webtools/plantcare/html/, accessed on 9 September 2022) (Lescot et al., 2002). Finally, TBtools was used to visualize the results, including CRE types and numbers.

### Chromosomal distribution, gene duplication, and synteny analyses of *EXP* genes

The melon genome was used to determine the chromosomal position of each *CmEXP* gene, and MapChart software was utilized to map the genes (Voorrips, 2002). According to the melon genome and its annotation file, *CmEXP* gene duplication was analyzed using TBtools. Thereafter, TBtools was employed to determine the nucleotide substitution parameters *Ka* (nonsynonymous) and *Ks* (synonymous), followed by the calculation of the *Ka*/*Ks* ratio. MCScanX in TBtools was used to generate the synteny relationships of the *CmEXP* genes between melon and the model plants (*Arabidopsis thaliana* and rice) and two Cucurbitaceae crops (cucumber and watermelon).

### Plant materials and RNA sequencing

All the experimental materials were planted in the Ledong Melon Experimental Base of Institute of Vegetables, Hainan Academy of Agricultural Sciences. A crack-resistant variety ‘Xizhoumi 17’ and a crack-susceptible variety ‘Xizhoumi 25’ were selected. At the fruit maturity stage, the exocarp (thickness < 0.5 cm) of non-cracked fruit of ‘Xizhoumi 17’ (N17), non-cracked (N25) and cracked fruit (C25) of ‘Xizhoumi 25’ were collected, and immediately frozen in liquid nitrogen. Then the samples were used for RNA-seq by Wuhan Metware Biotechnology Co., Ltd. (China).

### Expression profiles of *CmEXP* genes

The fragments per kilobase of exon model per million mapped fragments (FPKM) values of *CmEXP* genes were obtained from the melon peel transcriptome data of our laboratory (SRA accession number: SRP466450) to analyze the *CmEXP* gene expression patterns in fruit cracking. Based on the normalized data, heatmaps and Venn diagrams were generated using TBtools. ClusterProfiler (3.8.1) was used to perform Gene Ontology (GO) functional enrichment analysis of the DEGs.

The accuracy of RNA-seq data was validated by performing quantitative real-time polymerase chain reaction (qPCR) to confirm the DEGs. qPCR was performed using ChamQ™ Universal SYBR qPCR Master Mix (Vazyme, China). The PCR reaction system and amplification program were described in a previous study (Hu et al., 2021a), with *actin* as the internal reference gene. **Supplementary Table S1** lists the primer sequences for the selected *CmEXP* and reference genes.

### Prediction of the potential transcription factors and expression analysis

A 2.0-kb sequence upstream of the start codon of *CmEXPB1* served as the promoter sequence to predict its potential transcription factors. Then, using default parameters, this promoter sequence was submitted to the PlantRegMap database (http://plantregmap.gao-lab.org/) for *Arabidopsis thaliana*. BLAST was used to convert the predicted transcription factors into corresponding genes in the melon genome database. Subsequently, based on the transcriptome data, R functions were used to calculate Pearson’s correlation coefficients between all potential transcription factors and the above-mentioned melon genes. Finally, the criterion of Pearson’s correlation coefficient |r| of ≥0.8 was used to identify the potential transcription factors. Cytoscape v3.9.1 was employed for visualization.

Finally, many upregulated and downregulated transcription factors were randomly selected, and their expression patterns were investigated using qPCR with the ChamQ™ Universal SYBR qPCR Master Mix. **Supplementary Table S1** lists the gene primer sequences.

## Results

### *CmEXP* gene identification

Using HMMsearch, 33 EXP proteins with two conserved domains, namely DPBB_1 and Pollen_allerg_1 domains, were ascertained in the melon genome. Based on a previously reported standardized nomenclature (Gao et al., 2020; Liu et al., 2022a), all CmEXPs were categorized into four subgroups and sequentially named based on their chromosomal locations. The identified full-length sequences of CmEXP-encoded proteins were between 151 (CmEXLA3) and 281 (CmEXPA9) amino acids, with relative MWs between 16.82 kDa (CmEXLA3) and 31.10 kDa (CmEXPA9). Furthermore, the theoretical pI of the CmEXP family members was between 4.67 (CmEXLB2) and 10.21 (CmEXPA6), with 8.40 being the average value (**Table 1**). These findings indicate weakly alkaline properties.

**Table 1:** The physiochemical characteristics of *Expasin* genes identified in melon.

Gene name	Gene ID	Genomic location	ORF	AA	MW (kDa)	pI
CmEXPA1	MELO3C015695.2.1	chr1:27254883.27256106	693	230	25.32	8.25
CmEXPA2	MELO3C017181.2.1	chr2:24558233.24559836	762	253	26.69	8.32
CmEXPA3	MELO3C020005.2.1	chr3:19587131.19588643	738	245	26.09	7.31
CmEXPA4	MELO3C011350.2.1	chr3:24487268.24489057	783	260	28.42	9.67
CmEXPA5	MELO3C008552.2.1	chr5:12878722.12880678	738	245	26.28	8.9
CmEXPA6	MELO3C016517.2.1	chr6:26456017.26459659	726	241	26.54	10.21
CmEXPA7	MELO3C014013.2.1	chr6:32826523.32827535	798	265	29.01	9.95
CmEXPA8	MELO3C016062.2.1	chr7:18301863.18302845	717	238	26.69	8.38
CmEXPA9	MELO3C024488.2.1	chr8:8852281.8854081	846	281	31.10	9.27
CmEXPA10	MELO3C003134.2.1	chr8:30167309.30168740	783	260	28.08	9.68
CmEXPA11	MELO3C021999.2.1	chr9:1987889.1988962	735	244	26.29	8.62
CmEXPA12	MELO3C021619.2.1	chr9:4528480.4529659	795	264	28.51	9.08
CmEXPA13	MELO3C005613.2.1	chr9:21804988.21807018	786	261	28.39	9.62
CmEXPA14	MELO3C012108.2.1	chr10:2598904.2600499	750	249	26.30	8.88
CmEXPA15	MELO3C023866.2.1	chr10:6434224.6437071	786	261	28.26	9.76
CmEXPA16	MELO3C020143.2.1	chr10:9180157.9184262	762	253	27.35	7.99
CmEXPA17	MELO3C025907.2.1	chr11:20074684.20075750	780	259	27.96	9.79
CmEXPA18	MELO3C025785.2.1	chr11:26006896.26008101	753	250	27.90	8.37
CmEXPA19	MELO3C020626.2.1	chr12:1673411.1675196	783	260	27.83	9.51
CmEXPA20	MELO3C001993.2.1	chr12:25862084.25863125	762	253	27.00	9.04
CmEXPB1	MELO3C018743.2.1	chr1:2545552.2547928	741	246	26.61	8.48
CmEXPB2	MELO3C005962.2.1	chr6:240543.241811	825	274	28.88	5.6
CmEXPB3	MELO3C013606.2.1	chr11:18098616.18100562	813	270	29.35	8.1
CmEXLA1	MELO3C013289.2.1	chr1:15059036.15060534	804	267	29.73	7.85
CmEXLA2	MELO3C013292.2.1	chr1:15095678.15097481	798	265	29.36	7.85
CmEXLA3	MELO3C013293.2.1	chr1:15105675.15107263	456	151	16.82	5.91
CmEXLA4	MELO3C013294.2.1	chr1:15110617.15112109	810	269	29.96	8.87
CmEXLA5	MELO3C013295.2.1	chr1:15119061.15120990	795	264	29.33	7.85
CmEXLA6	MELO3C013296.2.1	chr1:15124725.15126667	810	269	30.16	6.03
CmEXLA7	MELO3C013299.2.1	chr1:15154867.15161011	795	264	29.69	9.13
CmEXLA8	MELO3C015050.2.1	chr2:8567550.8569383	801	266	30.03	9.54
CmEXLB1	MELO3C003336.2.1	chr4:386944.388976	768	255	28.47	6.64
CmEXLB2	MELO3C003371.2.1	chr4:659743.662048	771	256	27.92	4.67

### Phylogenetic analysis of EXP proteins

**Figure 1:**
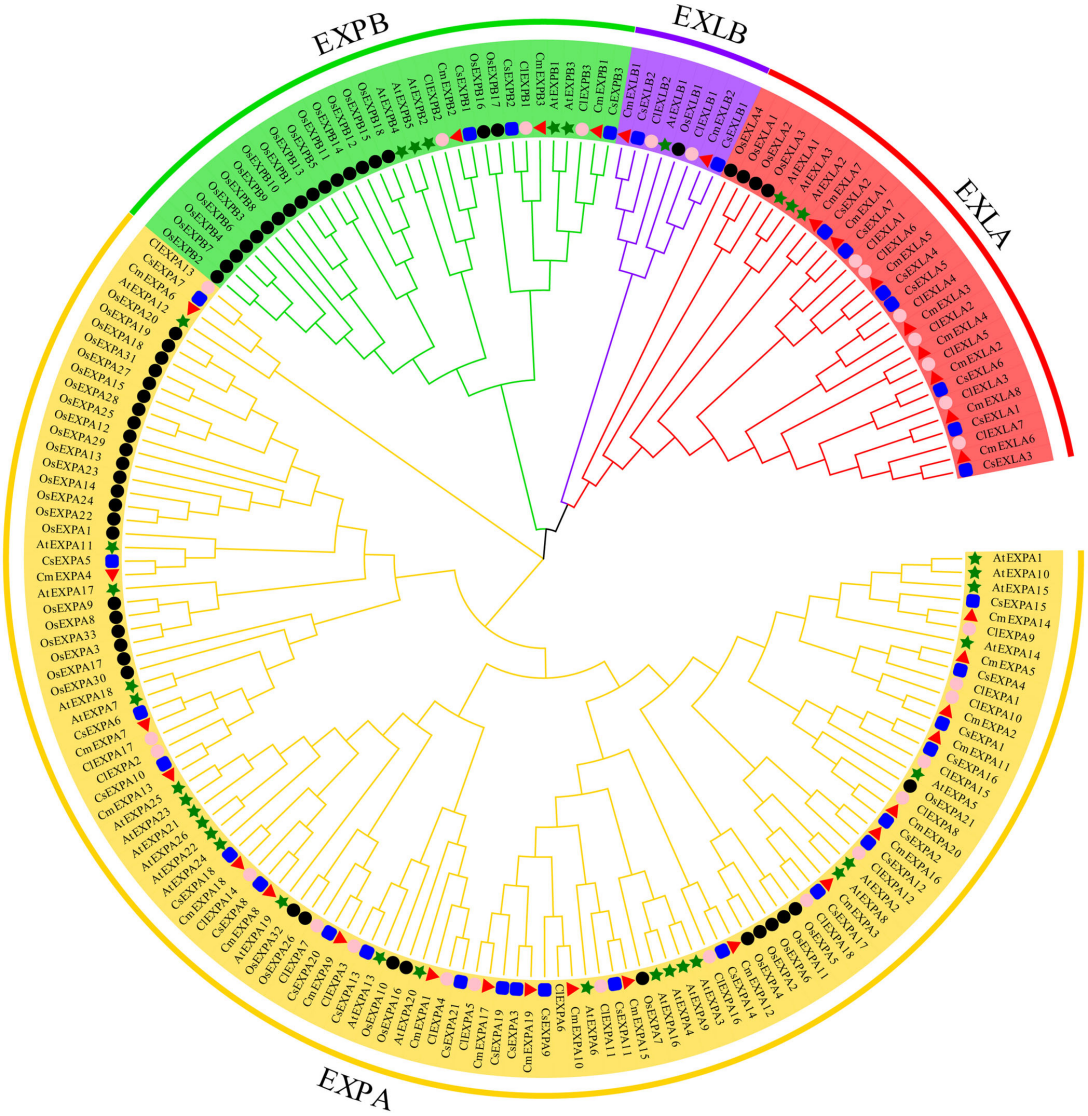
Phylogenetic analysis of the expansin (EXP) proteins from melon, cucumber, watermelon, *Arabidopsis*, and rice. MEGA X was used to construct the phylogenetic tree, and the maximum likelihood method with 1000 bootstrap replicates was used. Red triangles indicate melon EXP genes; green stars indicate *Arabidopsis thaliana* EXP genes; black circles indicate rice EXP genes; blue squares indicate cucumber EXP genes; and pink circles indicate watermelon EXP genes.

To understand the phylogenetic relationships of EXPs, ClustalW was used to align 33 CmEXPs, 33 EXP proteins from cucumber (CsEXPs), 30 from watermelon (ClEXPs), 35 from *Arabidopsis* (AtEXPs), and 56 from rice (OsEXPs). Thereafter, using MEGA X with 1000 bootstrap replications, an unrooted phylogenetic tree was constructed. The EXP proteins from the five species were divided into four subgroups: EXLA, EXLB, EXPA, and EXPB (**Figure 1**). The EXPA subgroup comprised the most members, with 20 CmEXPAs, followed by 8 CmEXLAs, 3 CmEXPBs, and 2 CmEXLB. This finding is consistent with the gene distribution in cucumber and watermelon (**Supplementary Table S2**). The CmEXPs in each phylogenetic tree branch were closely related to CsEXPs. This suggests that cucumber is the closest evolutionary relative of melon.

### Conserved motifs and gene structures of CmEXPs

**Figure 2:**
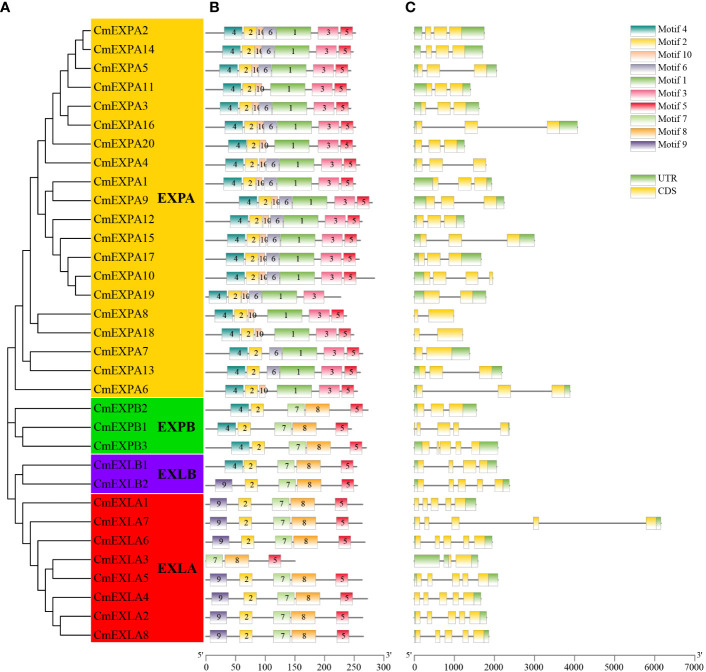
Phylogenetic relationships, protein domain architecture, and gene structures of *CmEXP* genes. **(A)** Phylogenetic relationship of 33 CmEXP proteins. MEGA X was used to construct the phylogenetic tree, and the maximum likelihood method with 1000 bootstrap replicates was used. **(B)** Conserved domain analysis of the CmEXP proteins. Different colored boxes indicate the different conserved motifs of the CmEXP proteins. **(C)** Analysis of the structure (exon–intron organization) of *CmEXP* genes. Gene Structure Display Server 2.0 was used to construct the gene structures. Red boxes, blue lines, and green boxes represent the coding sequences, introns, and untranslated regions, respectively. The scale bar is illustrated at the bottom.

The conserved motifs of CmEXP proteins were analyzed to elucidate the structural characteristics of the CmEXP gene family. Most proteins in the EXPA subgroup comprised motif 4-motif 2-motif 10-motif 6-motif 1-motif 3-motif 5. However, motif 6 was absent in CmEXPA6, CmEXPA8, and CmEXPA18, whereas motif 10 was absent in CmEXPA7 and CmEXPA13. Moreover, the proteins in the EXPB subgroup comprised motif 4-motif 2-motif 7-motif 8-motif 5. In the EXLA subgroup, only the CmEXLA3 protein lacked motifs 9 and 2 at the N-terminus, and the remaining members comprised motif 9-motif 2-motif 7-motif 8-motif 5. In the EXLB subgroup, CmEXLB1 comprised motif 4-motif 2-motif 7-motif 8-motif 5, whereas CmEXLB2 comprised motif 2-motif 2-motif 7-motif 8-motif 5 (**Figures 2A, B**). These results suggest that motif 10 is a unique conserved motif in the EXPA subgroup, in which motifs 7, 8, and 9 are absent. Collectively, these results indicate that the protein motifs are highly specific in different subgroups and that the protein motifs are highly conserved in each subgroup.

Gene structure analysis revealed 1–4 introns in the *CmEXP* gene family members (**Figure 2C**). In particular, most genes in the EXPA subgroup comprised two introns. However, *CmEXPA7*, *CmEXPA8*, *CmEXPA18*, and *CmEXPA19* comprised one intron, whereas *CmEXPA10* comprised three exons. Furthermore, both *CmEXPB1* and *CmEXPB3* comprised four introns, whereas *CmEXPB2* comprised two introns. In the EXLA subgroup, except for *CmEXLA3*, which comprised only one intron, all other *CmEXLA* genes comprised four exons. *CmEXLB1* and *CmEXLB2* comprised three and four introns, respectively. Moreover, the exon–intron structure features indicated gene structure similarities in the same subgroup.

### Chromosomal location of the *CmEXP* gene family

**Figure 3:**
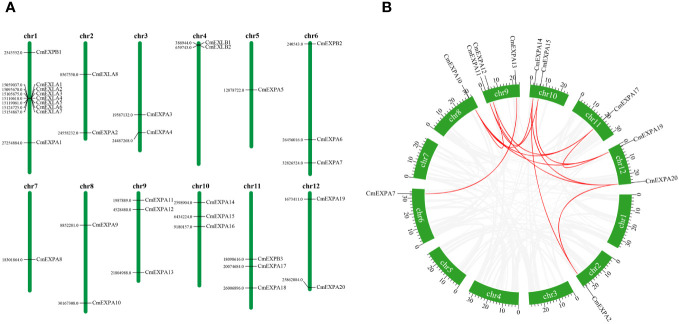
Chromosomal distribution and synteny analysis of *CmEXP* genes. **(A)** Chromosomal locations of *CmEXP* genes. Chromosome numbers are presented at the top of each chromosome. The numbers to the left of each chromosome represent the location of the *CmEXP* gene on the right. **(B)** Synteny relationships of the *CmEXP* gene family. Gray lines present the synteny blocks in the melon genome, whereas red lines between *CmEXP* genes present the duplication events that occurred in the *CmEXP* gene family.

Chromosomal location analysis of the *CmEXP* genes revealed that 33 genes were distributed across 12 chromosomes (**Figure 3A**). Each melon chromosome contained *CmEXP* genes. Chromosome 1 had the highest number of genes (9). Chromosomes 6, 9, 10, and 11 contained three *CmEXP* genes each, whereas chromosomes 2, 3, 4, 8, and 12 contained two *CmEXP* genes each. Chromosomes 5 and 7 only had one *CmEXP* gene each.

Next, gene distribution analysis in the different chromosome subgroups revealed the distribution of 20 *CmEXPA* genes across 11 chromosomes, with no genes identified on chromosome 4. Three *CmEXPB* genes were located on chromosomes 1, 6, and 11, whereas eight *CmEXLA* genes were only located on chromosomes 1 and 2. Chromosome 1 comprised 7 *CmEXLA* genes. Both *CmEXLB* genes were located on chromosome 4.

### Gene duplication and collinearity analysis of the *EXP* gene family

MCScanX was used to analyze the duplication events of *CmEXP* genes to reveal the amplification and evolutionary mechanisms of the *CmEXP* gene family. Thirteen duplication events involving 11 *CmEXP* genes were detected (**Figure 3B**): *CmEXPA14*/*CmEXPA20*, *CmEXPA15*/*CmEXPA19*, *CmEXPA14*/*CmEXPA2*, *CmEXPA15*/*CmEXPA10*, *CmEXPA14*/*CmEXPA11*, *CmEXPA17*/*CmEXPA19*, *CmEXPA17*/*CmEXPA10*, *CmEXPA17*/*CmEXPA12*, *CmEXPA20*/*CmEXPA2*, *CmEXPA19*/*CmEXPA10*, *CmEXPA20*/*CmEXPA11*, *CmEXPA7*/*CmEXPA13*, and *CmEXPA10*/*CmEXPA12*. The duplicated genes were primarily distributed on chromosomes 2, 6, 8, 9, 10, 11, and 12. The *Ka*/*Ks* ratio, which reflects the selection pressure during gene evolution, of the duplicated *CmEXP* genes was <1 (**Supplementary Table S3**). This suggests strong purifying selection and functional conservation during evolution.

**Figure 4:**
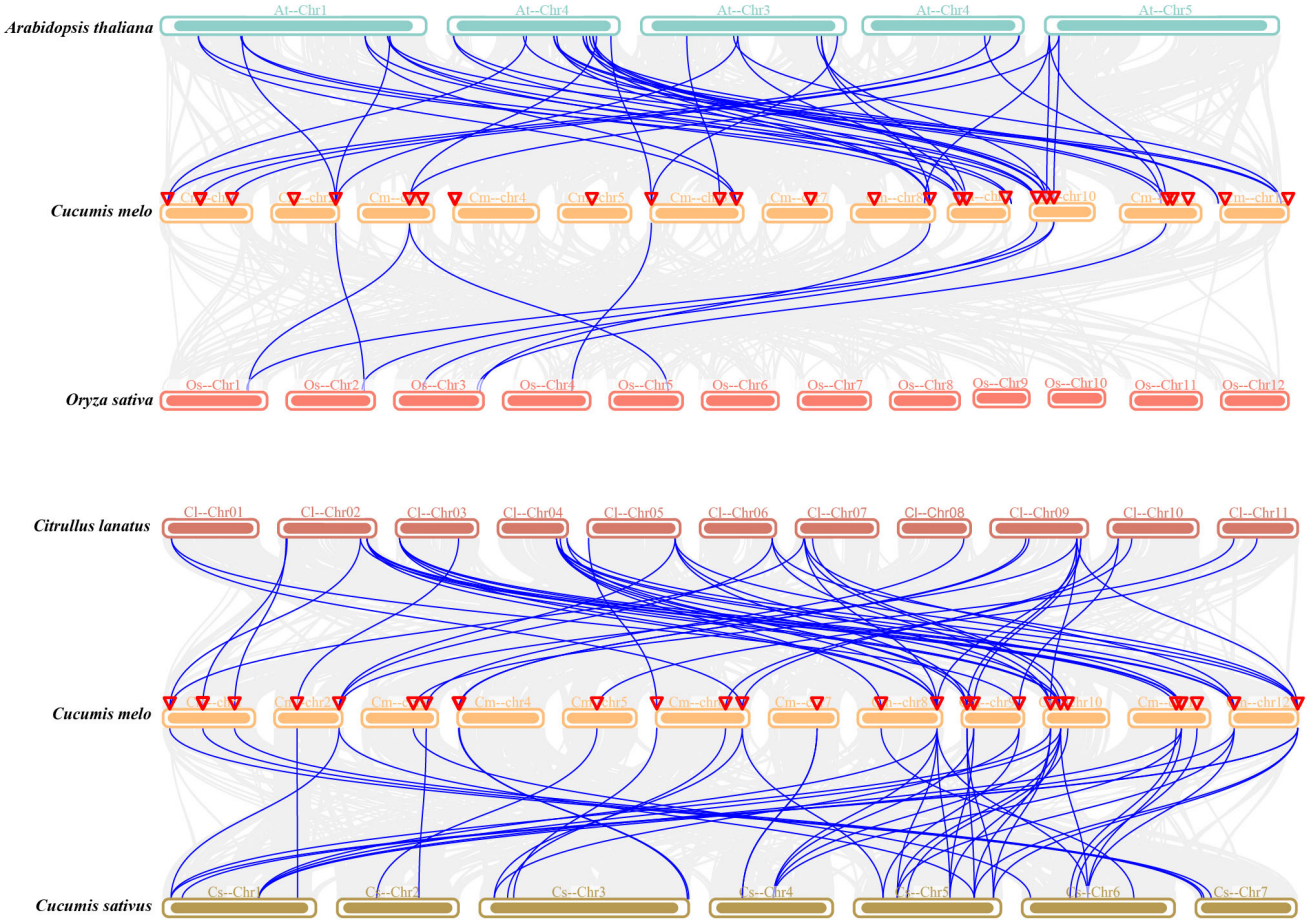
Synteny analysis of the *CmEXP* genes between *Cucumis melo* and four other plant species (*Arabidopsis thaliana*, rice, cucumber, and watermelon). Gray lines represent the significantly collinear blocks within and among the plant genomes, whereas blue lines represent the syntenic expansin gene pairs. The chromosome number is represented at the top of each chromosome. Red triangles indicate *CmEXP* genes.

Next, collinearity analysis of *CmEXP*s, *CsEXP*s, *ClEXP*s, *AtEXP*s, and *OsEXP*s was performed to elucidate the evolutionary relationship of the *CmEXP* gene family (**Figure 4**). Melon shared 50 collinear gene pairs with both cucumber and watermelon. Furthermore, 44 duplication events were detected between melon and *Arabidopsis*. However, melon had the fewest collinear genes (9) with rice, a monocotyledon (**Supplementary Table S4**).

### Detection of CREs in the promoter regions of *CmEXP*s

**Figure 5:**
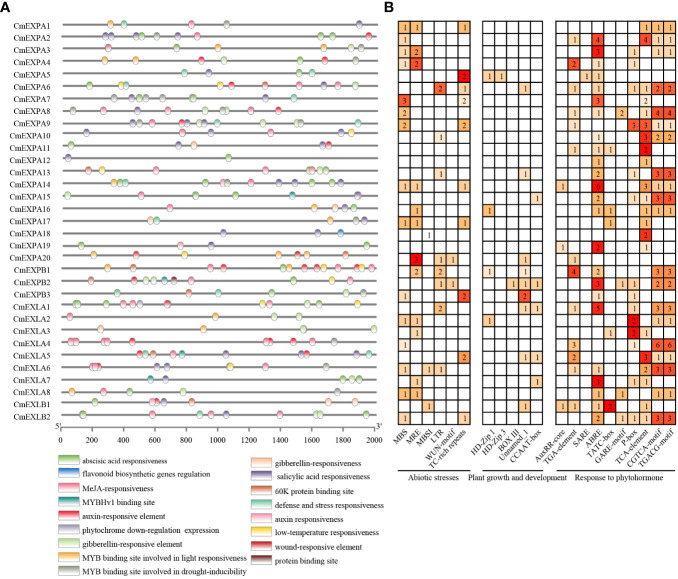
*Cis*-element analysis of the promoter regions of *CmEXP* genes. **(A)** Different *cis*-element types and their locations in each expansin gene are indicated using colored blocks. **(B)** The numbers of different promoter elements in *CmEXP* genes are represented using different colors and numbers. PlantCARE was used to deduce the numbers, types, and locations of the potential elements in the 2 kb upstream sequence of *CmEXP* genes.

To elucidate the mechanism underlying the transcriptional regulation of *CmEXP* genes, the 2.0 kb upstream sequence of the start codon (ATG) of *CmEXP* genes was selected as the promoter sequence to predict CREs. Twenty-one CREs exhibiting an uneven distribution pattern were identified in the promoter regions of *CmEXP* genes (**Figure 5**). These identified CREs were classified into three categories based on their functions: CREs related to abiotic stress (6 types), CREs related to plant growth and development (5 types), and CREs related to hormone responses (10 types). In terms of quantity, the CREs primarily participated in hormone pathways and abiotic stress responses. This suggests the vital role of *CmEXP* genes in hormone signaling and environmental stress adaptation. For abiotic stress, MBS was identified as the most abundant CRE (19 instances). It was noted to be present in the promoters of 15 *CmEXP* genes and annotated as an MYB-binding site associated with drought induction. For hormone responses, CREs were primarily involved in the following pathways: gibberellin, methyl jasmonate (MeJA), abscisic acid, auxin, and salicylic acid. ABRE, which is involved in abscisic acid responses, was noted to be the most abundant CRE (49 instances) (**Figure 5**; **Supplementary Table S5**).

### RNA-seq analysis of *EXP* genes in the fruit peel of netted melon

**Figure 6:**
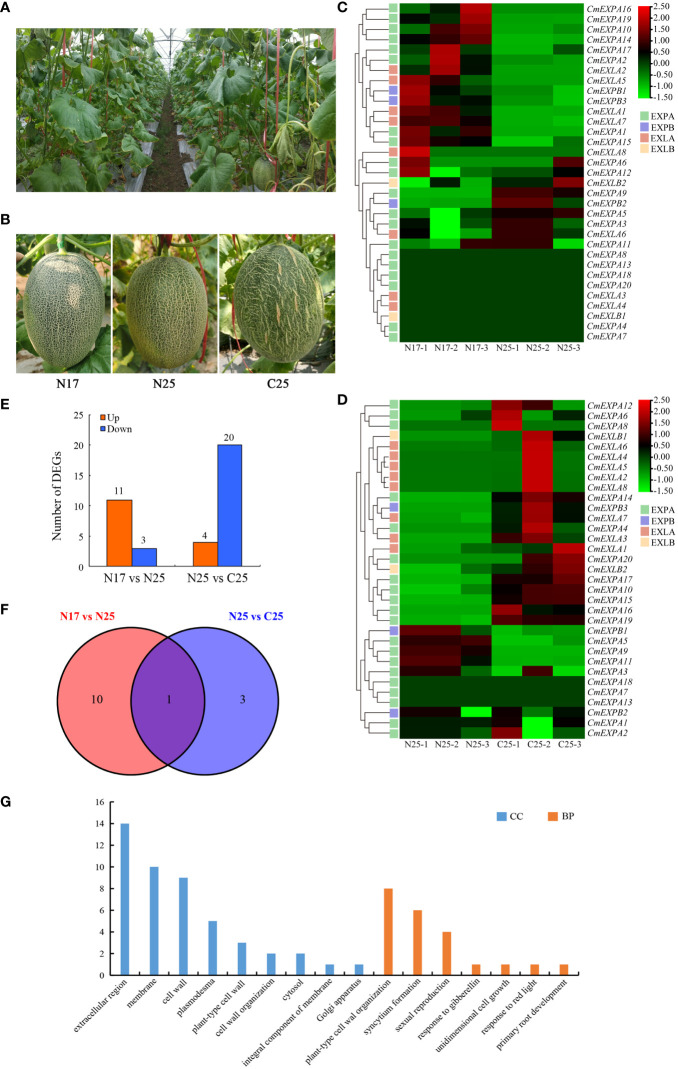
RNA-seq analysis of the *CmEXP* gene expression patterns. **(A)** Photographs of the fruits of netted melons in the field. **(B)** Different fruit peel types of netted melon. N17 indicates the non-cracking fruit peel of the cracking-resistant variety ‘Xizhoumi 17’, N25 indicates the non-cracking fruit peel of the cracking-susceptible variety ‘Xizhoumi 25’, and C25 indicates the cracking fruit peel of ‘Xizhoumi 25’. **(C)** Heatmap illustrating the *CmEXP* genes in the non-cracking fruit peels of ‘Xizhoumi 17’ (N17) and ‘Xizhoumi 25’ (N25). **(D)** Heatmap illustrating the *CmEXP* genes in the non-cracking (N25) and cracking (C25) fruit peels of ‘Xizhoumi 25’. **(E)** The number of upregulated or downregulated *CmEXP* genes is illustrated in **(C)** and **(D)**. The expression change of the *CmEXP* with a log2FC (FC: fold change) value > 1 is designated as upregulated, whereas the value < -1 as downregulated. **(F)** Venn diagram of the upregulated differentially expressed genes (DEGs) shared among the N17 *versus* N25 and N25 *versus* C25 groups. **(G)** Gene Ontology functional annotation of the upregulated DEGs.

To elucidate the involvement of *CmEXP* genes in netted melon fruit peel cracking, the expression patterns of 33 *CmEXP* genes in the fruit peel (including cracked and non-cracked peel) of the crack-resistant netted melon variety ‘Xizhoumi 17’ and the crack-susceptible netted melon variety ‘Xizhoumi 25’ (**Figures 6A, B**) were analyzed using transcriptome data. *CmEXP* gene expression was different in the different fruit peel varieties. When comparing the non-cracked fruit peels of the crack-resistant variety ‘Xizhoumi 17’ (N17) and the crack-susceptible variety ‘Xizhoumi 25’ (N25), 23 *CmEXP* genes were observed to be expressed in both varieties, with significant differences in the expression of 14 *CmEXP* genes (*P* < 0.05). Among them, 11 *CmEXP* genes, namely, *CmEXPA2*, *CmEXPA10*, *CmEXPA14*, *CmEXPA16*, *CmEXPA19*, *CmEXPB1*, *CmEXPB3*, *CmEXLA1*, *CmEXLA2*, *CmEXLA5*, and *CmEXLA7*, were significantly upregulated in the non-cracked fruit peel of N17, whereas 3 *CmEXP* genes, namely, *CmEXPA5*, *CmEXPA9*, *CmEXPB2*, were significantly downregulated (**Figures 6C, E**; **Supplementary Table S6**). Furthermore, the *CmEXP* gene expression patterns in the non-cracked (N25) and cracked (C25) fruit peels of the crack-susceptible variety ‘Xizhoumi 25’ were analyzed. Thirty *CmEXP* genes were expressed in both tissues, with significant differences in the expression of 24 *CmEXP* genes (*P* < 0.05). Among them, 4 *CmEXP* genes, namely, *CmEXPA5*, *CmEXPA9*, *CmEXPA11*, and *CmEXPB1*, were significantly upregulated in N25, whereas 20 *CmEXP* genes, namely, *CmEXPA4*, *CmEXPA6*, *CmEXPA8*, *CmEXPA10*, *CmEXPA12*, *CmEXPA14*, *CmEXPA15*, *CmEXPA16*, *CmEXPA17*, *CmEXPA19*, *CmEXPA20*, *CmEXPB3*, *CmEXLA2*, *CmEXLA3*, *CmEXLA4*, *CmEXLA5*, *CmEXLA6*, *CmEXLA7*, *CmEXLA8*, and *CmEXLB1*, were significantly downregulated in N25 (**Figures 6D, E**; **Supplementary Table S6**).

Next, GO enrichment analysis of the upregulated genes in the N17_vs_N25 and N25_vs_C25 groups was performed to elucidate the functional and regulatory pathways of the DEGs. Two categories were identified: biological process (BP) and cellular component (CC) (**Figure 6G**). In the BP category, the most enriched genes were involved in plant-type cell wall organization (8 genes), syncytium formation (6 genes), and sexual reproduction (4 genes). In the CC category, the most enriched genes were involved in the extracellular region (14 genes), membrane (10 genes), and cell wall (9 genes).

### Validation of the expression patterns of RNA-seq data using qPCR

**Figure 7:**
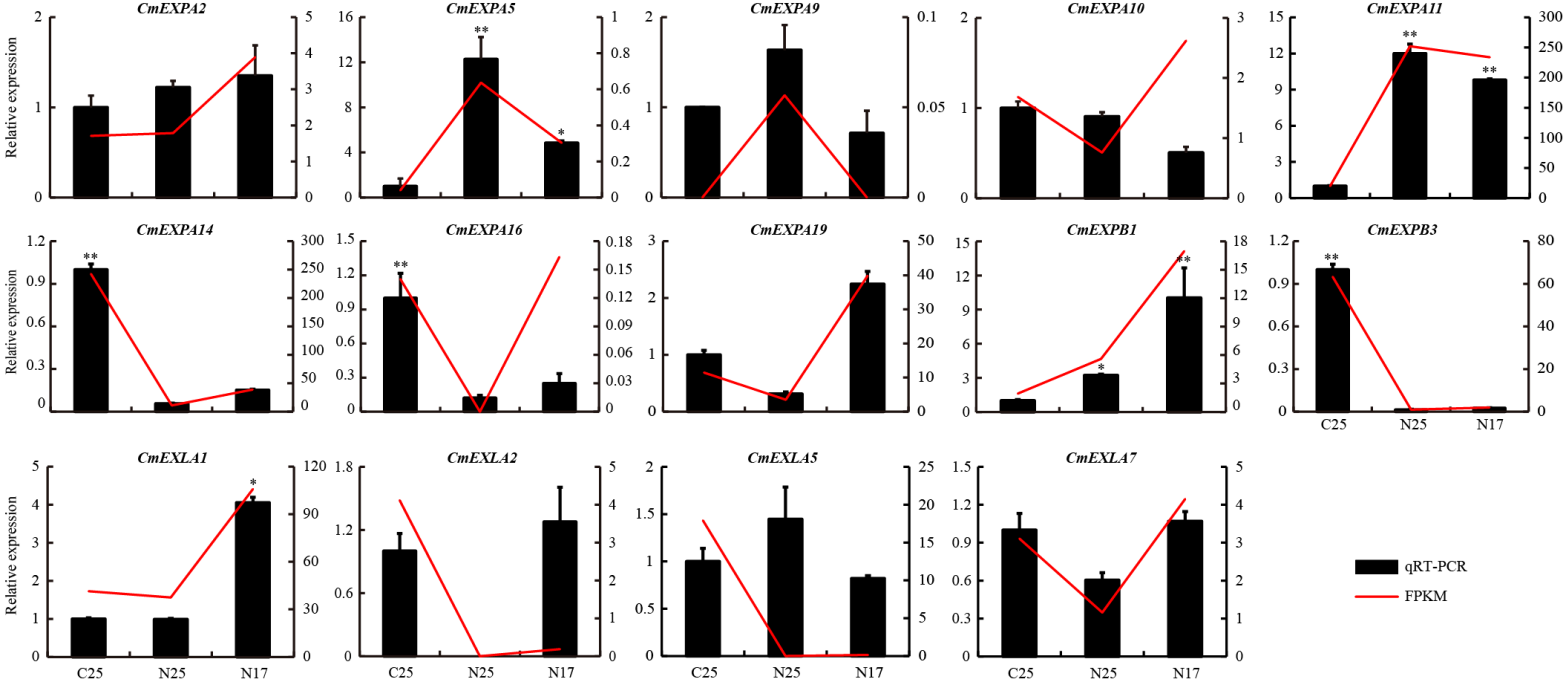
Validation of the upregulated *CmEXP* genes via quantitative real-time polymerase chain reaction. Error bars represent the standard deviation of three independent replicates. Asterisks (* and **) denote significant differences at P < 0.05 and < 0.01, respectively.

The transcriptome data revealed that the expression of four *CmEXP* genes (*CmEXPA5*, *CmEXPA9*, *CmEXPA11*, and *CmEXPB1*) was significantly upregulated in the non-cracked fruit peel (N25) than in the cracked fruit peel (C25) of the variety ‘Xizhoumi 25’ (**Figure 6D**; **Supplementary Table S6**). Moreover, a comparison of the non-cracked fruit peel of ‘Xizhoumi 25’ (N25) with the non-cracked fruit peel of ‘Xizhoumi 17’ (N17) revealed that 11 *CmEXP* genes were significantly upregulated in N17, a crack-resistant variety (**Figure 6C**; **Supplementary Table S6**). To additionally elucidate the gene expression patterns, qPCR was performed to validate the upregulated genes. Consistent trends were observed between qPCR and RNA-seq (**Figure 7**), confirming the reliability of the transcriptome sequencing results.

### Prediction and screening of the transcription factors of *CmEXPB1* and validation of gene expression

**Figure 8:**
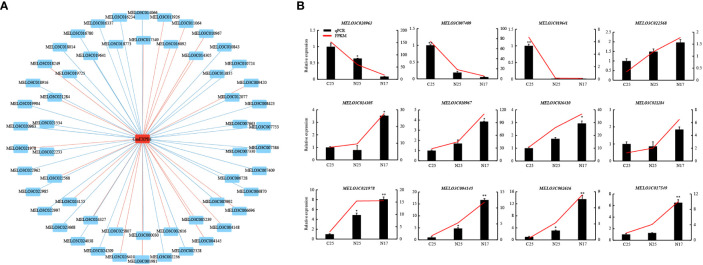
Prediction and screening of the transcription factors of *CmEXPB1*. **(A)** Regulatory network of *CmEXPB1* and its corresponding candidate transcription factors (TFs) using RNA-seq data. Significant positive and negative correlations are indicated using red and green lines, respectively. **(B)** RNA-seq and quantitative real-time PCR showing the expression patterns of the candidate TFs. Asterisks (* and **) denote significant differences at P < 0.05 and < 0.01, respectively.

A comparison of the two experimental groups revealed that only one gene was significantly upregulated in both groups (**Figure 6F**). qPCR was performed to verify this finding, and *CmEXPB1* was identified as the gene (**Figure 6F**). This suggests the vital role of *CmEXPB1* in the crack resistance of netted melon fruit peel. Next, to understand the transcriptional regulation mode of *CmEXPB1*, the transcription factors that bind to the promoter region of *CmEXPB1* were predicted. In total, 949 transcription factor binding sites were predicted in the 2.0 kb sequence upstream of the ATG codon of the promoter region of *CmEXPB1* (**Supplementary Table S7**). Thereafter, correlation analysis (Pearson’s correlation coefficient |r| > 0.8 and *P* < 0.05) of the predicted transcription factors and *CmEXPB1* expression led to the identification of 56 transcription factors. Among them, 21 transcription factors exhibited a positive correlation, whereas 35 exhibited a negative correlation (**Figure 8A**; **Supplementary Table S8**). These transcription factors may participate in *CmEXPB1* expression regulation and the crack resistance responses of netted melon fruit peel. Statistical analysis revealed that these 56 transcription factors belong to 21 transcription factor families: 15 MYB, 7 WRKY, 5 bHLH, 5 ERF, 5 HD-ZIP, 2 bZIP, 2 C2H2, and 2 Trihelix transcription factors, and the remaining 13 transcription factor families comprised only one member each (**Supplementary Table S8**).

To investigate the expression patterns of the candidate transcription factors, RNA-seq and qPCR were performed to determine the expression of 12 transcription factors. The RNA-seq data were consistent with those of qPCR (**Figure 8B**). Among them, nine transcription factors were upregulated, whereas three were downregulated in N17 compared with C25, and their expression trends were consistent or opposite to the *CmEXPB1* expression pattern (**Figure 7**). These results suggest that these transcription factors regulate *CmEXPB1* expression positively or negatively.

## Discussion

Fruit cracking during growth and development is a challenging issue in fruit production. Previous studies have investigated fruit cracking from the perspectives of anatomical structure, fruit shape, and growth characteristics. For example, in the crack-susceptible tomato variety, the maximum pressure on the fruit peel is near the calyx (Peet, 1992). Larger cherry tomatoes are more prone to cracking than smaller ones (Sekse, 1987; Sekse, 2008). Additionally, there is a positive correlation between the cracking rate of grapefruit and its shape index (longitudinal diameter–transverse diameter ratio), and crack-susceptible grape varieties have larger transverse and longitudinal diameters (Zhang et al., 2020). Regarding growth characteristics, rapid fruit growth in tomatoes promotes cracking (Khadivi-Khub, 2015). Furthermore, the absolute growth rate of the transverse diameter is higher than that of the longitudinal diameter in crack-susceptible tomato varieties, whereas the absolute growth rates of the two diameters are extremely similar in crack-resistant varieties (Yang et al., 2016). However, the molecular mechanisms underlying the crack resistance of fruit peels remain unclear. Transcriptome sequencing of the resistant and susceptible-cracking watermelon peel revealed that several genes play roles in watermelon cracking resistance (Jiang et al., 2019). Transcriptome sequencing of the cracking susceptible and the cracking tolerant sweet cherry cultivars revealed significant changes in gene expression related to the biosynthesis of expansins, aquaporins, abscisic acid, ethylene, etc. (Michailidis et al., 2021). These results will contribute to the understanding and development of the molecular basis of fruit cracking and plant breeding.

In plant cells, the cell wall is an essential and unique structure that regulates cell shape and size, provides mechanical support and rigidity to cells, and functions as the first line of defense against external environmental stimuli (Liu et al., 2018). The decomposition, modification and composition of cell wall can affect the performance of pericarp and is an important factor in determining the sensitivity of fruit cracking (Bruggenwirth and Knoche, 2017; Yang et al., 2016; Jiang et al., 2019). Therefore, enzymes and proteins that regulate cell wall metabolism, such as polygalacturonase (PG), β-galactosidase (β-Gal), and expansin (EXP), are particularly important in the resistance to fruit cracking (Yu et al., 2020). EXPs can induce cell wall loosening and is crucial for regulating the cracking process of fruit peels (Wang et al., 2006; Kasai et al., 2008; Liu et al., 2022b).

Previous studies have elucidated the *EXP* gene family in many plants, with the systematic analysis of the *EXP* gene family in various plants, including *Arabidopsis* (model plant) (Sampedro et al., 2006), crops such as rice (Sampedro et al., 2006), vegetable crops such as tomato (Jiang et al., 2019) and pepper (Liu et al., 2022b), and fruit crops such as apple (Kasai et al., 2008), litchi (Li et al., 2014), and pomegranate (Xu et al., 2023). Subsequent studies have revealed a close relationship between EXPs and fruit cracking. Fruit cracking significantly hampers fruit appearance, facilitates pathogen invasion, and results in significant economic losses (Wang et al., 2021). During ripening, netted melon is prone to fruit cracking (Hu et al., 2021b). Therefore, there is an urgent and vital need to elucidate the role of EXP proteins in netted melon. Herein, 33 *EXP* genes were identified in the genome of netted melon, similar to the number identified in pumpkin (33) (Gao et al., 2020) and watermelon (30) (Liu et al., 2022a). Thirty-five *EXP* genes have been identified in the model plant *Arabidopsis*, whereas 56 *EXP* genes have been identified in rice, a monocot plant (Sampedro et al., 2006). This suggests the different replication methods of the *EXP* gene family in monocots and dicots, which is possibly associated with plant evolution (Cosgrove et al., 2002). Phylogenetic analysis revealed that the EXP gene family in plants can be divided into four subgroups: EXPA, EXPB, EXLA, and EXLB (**Figure 1**), which are present in the EXP gene families of different plants (Sampedro et al., 2006; Liu et al., 2022a; Xu et al., 2023). A comparison of the number of members among the four subgroups within the EXP gene families in the five different plant species revealed that the EXPA subgroup comprises more members. Furthermore, the EXPB subfamily in monocot rice has significantly more members than the other four dicot plants (**Figure 1**; **Supplementary Table S2**). This finding further suggests evolutionary differences in the gene replication and amplification of *EXP* genes between monocots and dicots, with monocots having more gene expansion and retention during gene replication (Zhu et al., 2014). Moreover, evolutionary tree analysis revealed that in the two largest subgroups, i.e., EXPA and EXPB, the major branches generally contain *EXP* genes from different species, whereas in some small branches, only *EXP* genes from the same species are present. These findings indicates that *EXP* genes undergo amplification before species differentiation. Similar results have been observed in tobacco (Ding et al., 2016) and pomegranate (Xu et al., 2023). Conserved protein motif analysis demonstrated that the CmEXP proteins in the same subgroup exhibit similar conserved elements. Gene structure analysis revealed that there are 1–4 introns in *CmEXP* genes, and this finding is in line with that of other plants (Dal Santo et al., 2013; Xu et al., 2023). These structural features suggest that the *CmEXP* genes in the same subgroup share similar structural characteristics and have been subjected to gene duplication events during evolution.

Gene duplication events are essential for genome rearrangement and amplification (Vision et al., 2000). The analysis of the duplication events in the gene family revealed that 13 gene pairs were duplicated in the *CmEXP* gene family (**Figure 3B**). This indicates that species-specific gene duplication has strongly affected this family’s evolution. *Ka*/*Ks* analysis revealed that the duplicated genes have undergone strong purification selection pressure (**Supplementary Table S3**), indicating the highly conserved evolutionary pattern of *CmEXP* genes. The estimated duplication time for paralogous genes indicates that all paralogs, except for *CmEXPA15*/*CmEXPA19*, are ancient and range from 337 to 955 million years ago (**Supplementary Table S3**). Homology analysis of the *EXP* gene families in different species revealed that *CmEXP* genes have 50 homologous genes in cucumber and melon, 44 homologous genes in *Arabidopsis*, and only 9 homologous genes in rice (**Figure 4**). These results suggest evolutionary variation between monocots and dicots in terms of *EXP* genes, with higher homology in closely related species (melon and pumpkin, and melon and watermelon).

CREs help genes respond to growth, development, or environmental adaptation. Previous research has demonstrated that the upstream promoter region of *CmEXP* genes contains elements involved in plant growth, abiotic stress, and hormone induction, and this finding is consistent with that observed for other plant *EXP* gene promoters (Xu et al., 2023), indicating that the CREs are conserved in the *EXP* gene promoters across species. The *CmEXP* gene promoter contains multiple stress-related CREs (**Figure 4**), suggesting that these genes participate in abiotic stress responses. Moreover, previous studies revealed that plant *EXP* genes can regulate abiotic stress. *EaEXPA1* overexpression improves drought tolerance in transgenic sugarcane (Narayan et al., 2021). Furthermore, in transgenic *Arabidopsis*, the *Osmanthus fragrans* OfEXLA1 can improve salt and drought tolerance, which is regulated by the abscisic acid signaling pathway (Dong et al., 2023). When the wild peanut *AdEXLB8* gene is heterogeneously expressed in tobacco, it leads to cell wall reorganization, enhancing both drought tolerance and resistance to *Sclerotinia sclerotiorum* and *Meloidogyne incognita* (Brasileiro et al., 2021). In addition, the promoter region contains multiple hormone-related CREs (**Figure 5B**), indicating that hormone signaling pathways regulate *CmEXP* gene functions. A recent study on jujube fruit cracking revealed that abscisic acid and MeJA treatment induce fruit cracking (Liu et al., 2023), suggesting that these two hormones negatively regulate jujube fruit cracking. The RNA-seq analysis of crack-resistant and crack-susceptible jujube varieties revealed that the most DEGs (including *EXPA* genes) are enriched in the cell wall synthesis pathway (Liu et al., 2023). This indicates the role of *EXP* genes in jujube fruit cracking.

To elucidate the role of *CmEXP* genes in the crack resistance of netted melon fruit, transcriptome sequencing on different fruit peel types was conducted. The *CmEXP* gene expression patterns were different between the non-cracked and cracked fruit peels of the crack-susceptible and crack-resistant netted melon varieties (**Figures 6C, D**). When comparing N17 with N25, 14 differentially expressed *CmEXP* genes were identified; in contrast, when comparing N25 and C25, 24 differentially expressed *CmEXP* genes were identified (**Figure 6E**). Fewer significant changes were observed in *CmEXP* gene expression in the non-cracked fruit peel of the different varieties; however, the *CmEXP* genes exhibited a more evident varying trend in the non-cracked and cracked fruit peels of the same variety. This indicates the importance of *CmEXP* genes in the crack resistance of netted melon. To identify the key *CmEXP* genes involved in crack resistance, the upregulated genes in both datasets were analyzed. Only one gene, i.e., *CmEXPB1*, was consistently upregulated (**Figure 6F**). Therefore, *CmEXPB1* may play a vital role in the crack resistance of netted melon, and transgenic or gene silencing techniques can be used to validate its function (Liu et al., 2022b). Transcription factors are essential for regulating gene function (Donovan and Larson, 2022). Previous studies have demonstrated that various transcription factors regulate *EXP* gene expression. The banana transcription factor MaERF11 can inhibit *MaEXP2*, *MaEXP7*, and *MaEXP8* gene expression, thereby regulating banana ripening (Han et al., 2016). Furthermore, the transcription factor MaBSD1 regulates *MaEXP1* and *MaEXP2* expression in response to banana ripening (Ba et al., 2014). ZmNAC11 and ZmNAC29, two NAC transcription factors in maize, can activate *ZmEXPB15* expression, thereby improving grain size and weight by regulating nuclear elimination (Sun et al., 2022). To elucidate the transcriptional regulatory mechanism of *CmEXPB1*, we predicted its interacting transcription factors and identified 56 transcription factors that may regulate *CmEXPB1* expression positively or negatively (**Figure 8A**). Nevertheless, additional research is warranted to validate the functions of these transcription factors.

## Conclusion

Herein, 33 *CmEXP* genes were identified. Phylogenetic tree analysis of CmEXPs, CsEXPs, ClEXPs, AtEXPs, and OsEXPs revealed that CmEXP proteins can be categorized into four subfamilies: EXPA (20 members), EXPB (8 members), EXLA (3 members), and EXLB (2 members). The motifs and gene structures of the members of these subfamilies are highly conserved. CRE analysis in the promoter region suggests that *CmEXP* genes respond to development and stress. Furthermore, using transcriptome data, the *CmEXP* gene expression patterns in the non-cracked and cracked fruit peels of netted melon were analyzed, identifying 14 upregulated genes. Among them, *CmEXPB1* was consistently upregulated in both datasets, indicating its potential role in the crack resistance of netted melon. Transcription factor prediction led to the identification of 21 and 35 positive and negative regulators, respectively, potentially involved in the regulation of *CmEXPB1* expression. The above-mentioned findings enrich our understanding of the *CmEXP* gene family and suggest that *CmEXPB1* is a candidate gene involved in regulating the crack resistance of netted melon.

## Data availability statement

The datasets presented in this study can be found in online repositories. The raw sequencing reads can be retrieved from National Center for Biotechnology Information (NCBI) database with the accession number PRJNA1026294.

## Author contributions

YH: Data curation, Formal analysis, Investigation, Methodology, Project administration, Resources, Software, Writing – original draft. YL: Formal analysis, Investigation, Methodology, Software, Writing – original draft. BZ: Investigation. WH: Formal analysis. JC: Investigation. FW: Formal analysis. YC: Investigation. MW: Funding acquisition, Resources, Supervision, Writing – review & editing. HL: Supervision, Validation, Writing – review & editing. YZ: Conceptualization, Formal analysis, Funding acquisition, Investigation, Methodology, Project administration, Resources, Software, Supervision, Validation, Visualization, Writing – original draft, Writing – review & editing.
