# Supplementary material for: Genome-wide identification of the expansin gene family in netted melon and their transcriptional responses to fruit peel cracking
Source: Front Plant Sci. 2024 Jan 23;15:1332240. doi: 10.3389/fpls.2024.1332240 (PMC10846642; doi:10.3389/fpls.2024.1332240)
Supplement: Supplementary file 1 [file Table_1.docx]

Supplementary Material

Genome-wide identification of the expansin gene family in netted melon and their transcriptional responses to fruit peel cracking

**Yanping Hu^1, 2, 3,^** ^†^**, Yuxin Li^1, 3,^** ^†^**, Baibi Zhu^3^, Wenfeng Huang^3^, Jianjun Chen^2^, Feng Wang^2^, Yisong Chen^2, 3^, Min Wang^2, 3, *^, Hanggui Lai^1, *^, Yang Zhou^1, *^**

*** Correspondence:** Yang Zhou: zhouyang@hainanu.edu.cn; Hanggui Lai: laihanggui8938@163.com; Min Wang: 13005022331@163.com

^†^These authors contributed equally to this work

**Supplementary Table S1** Primers used in this study.

**Supplementary Table S2** Number of *Expansin* genes in five plant species.

**Supplementary Table S3** Estimated *Ka*/*K*s ratios of the duplicated expansin genes in *Cucumis melo*.

**Supplementary Table S4** Collinearity of expansin genes in *Cucumis melo* (Cm) and *Arabidopsis thaliana* (At), *Oryza sativa* (Os), *Cucumis sativus* (Cs) and *Citrullus lanatus* (Cl).

**Supplementary Table S5** *Cis*-acting elements analysis of *CmEXP* gene promoters.

**Supplementary Table S6** Expression of *CmEXP* genes in the fruit peels of different netted melon varieties (FPKM).

**Supplementary Table S7** TFs were predicted to bind in the promoter region of *CmEXPB1*.

**Supplementary Table S8** The TFs that showed high Pearson correlation coefficients with *CmEXPB1* gene.
